# A benchmark dataset for binary segmentation and quantification of dust emissions from unsealed roads

**DOI:** 10.1038/s41597-022-01918-x

**Published:** 2023-01-05

**Authors:** Asanka De Silva, Rajitha Ranasinghe, Arooran Sounthararajah, Hamed Haghighi, Jayantha Kodikara

**Affiliations:** 1 ARC Industrial Transformation Research Hub (ITRH) — SPARC Hub, Department of Civil Engineering, Monash University, Clayton Campus, Clayton, VIC 3800 Australia; 2 National Research and Development Laboratory, Downer EDI Works, Somerton, VIC 3061 Australia

**Keywords:** Environmental sciences, Civil engineering

## Abstract

The generation of reference data for machine learning models is challenging for dust emissions due to perpetually dynamic environmental conditions. We generated a new vision dataset with the goal of advancing semantic segmentation to identify and quantify vehicle-induced dust clouds from images. We conducted field experiments on 10 unsealed road segments with different types of road surface materials in varying climatic conditions to capture vehicle-induced road dust. A direct single-lens reflex (DSLR) camera was used to capture the dust clouds generated due to a utility vehicle travelling at different speeds. A research-grade dust monitor was used to measure the dust emissions due to traffic. A total of ~210,000 images were photographed and refined to obtain ~7,000 images. These images were manually annotated to generate masks for dust segmentation. The baseline performance of a truncated sample of ~900 images from the dataset is evaluated for U-Net architecture.

## Background

Unsealed roads are the largest component of the road network in many countries, including Australia, New Zealand and South Africa, where unsealed roads amount to approximately 60%, 40%^1^ and 75%^2^ of all roads, respectively. Dust emission from these roads is a serious issue, as it has adverse health and environmental impacts^3,4^. Furthermore, the generated dust cloud reduces road visibility^5^ leading to traffic hazards^6,7^. Dust is essentially the loss of material from the road surface, which is an indication of the degree of deterioration of an unsealed road^8^. In order to determine the most appropriate maintenance strategy and minimise dust emissions, the dust generated needs to be quantified. Multiple methods exist to measure dust emissions from unsealed roads. The AP-42 dust model developed by the United States Environmental Protection Agency (USEPA) estimates the quantity of particles with diameters less than or equal to 10 µm (*PM*_10_)^9^; however, this empirical model shows discrepancies with *in-situ* dust measurements^10^. There are various field-based methods for physical measurement of dust in terms of mass or volumetric density. These methods vary from vacuum pumps to light-scattering systems^11–14^. Recently, several research groups have investigated the performance of different machine learning (ML) methods in detecting dust in large-scale dust events observed by satellites utilising different dust spectrum signatures such as empirical thresholds, dust false colour imaging, dust index^15–17^, deep blue algorithm^18^ and K-means clustering^19^. However, the applicability of these methods to localised dust events, such as dust emissions from unsealed roads is still questionable. Further, the sensitivity of these methods to spectral bands used during training has not yet been examined. In a recent study, encoder-decoder ML models were used to perform dust segmentation for satellite images of dust storms on Mars^20^. However, more advanced, recently-developed ML models with more components than encoder-decoder models could be used for more accurate dust segmentation. Another study used smartphones to gather images of dust emissions from unsealed roads and collected visual indicators for further image analysis using different filters; however, only dust classification and dust severity were reported, and the dataset is not publicly available^21^. Researchers have also explored different classification methods for dust detection in satellite data, such as vector machines and random forests^22^, and found that the algorithms perform better than the statistical techniques^23^. Further, a classification study was carried out on dust emissions in a coal preparation plant by localising particle overlapping regions and feature learning through a discriminatory network^24^. Unlike classification techniques, semantic segmentation has an advantage as it could distinguish the boundary of the object and the object area that requires identification, providing semantic segmentation with an upper hand on quantification problems. A reliable dust measurement technique should be able to recognise different sizes of particles. Image processing and analysis cannot do this directly, as most particle sizes are too small to capture in a single pixel. However, if the particles combine together (e.g. in a dust cloud), pixel-wise dust detection of an image becomes possible^25^. Past studies have used supervised machine learning methods to recognise dust pixels in satellite images. Trained models are then used to determine whether an observed pixel on the satellite image is dust^26–28^.

The application of supervised machine learning tools for the detection of dust from unsealed roads is still very new. The major limitation of supervised machine learning is the requirement of a large training set of images^29^ from unsealed roads in which dust pixels are labelled. The manual pixel-wise labelling of images is labour-intensive and very time-consuming^30^. However, a dataset consisting of raw and labelled images remains essential for use in machine learning techniques to accurately identify and quantify dust. Our dataset offers ~7,000 ground truth images and ~7,000 annotated images in a benchmark set, enabling its use for training deep learning models to recognise the dust pixels on unsealed roads.

## Methods

Field experiments were designed to gather images of vehicle-induced road dust with their corresponding dust concentrations. Ten unsealed road segments in Victoria, Australia were selected for the conduct of the experiments.

### Test locations

**Fig. 1:**
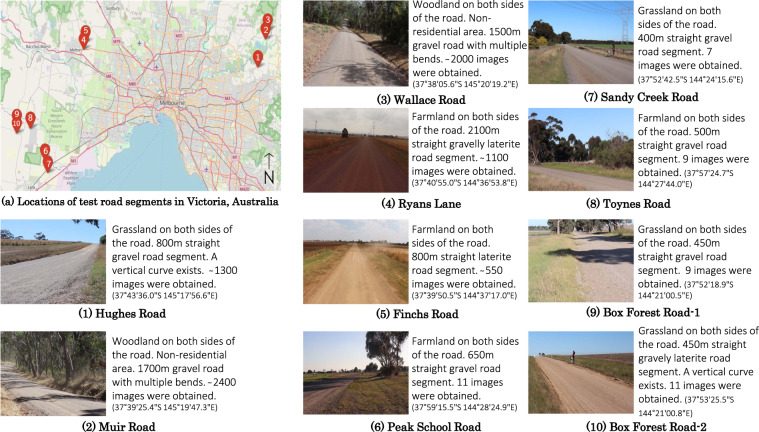
Details of the test road segments and the nature of the surface of each road segment. Images from Hughes Road, Muir Road, Wallace Road, Ryan’s Lane and Finchs Road were used for training and the images from Peak School Road, Sandy Creek Road, Toynes Road, Box Forest Road-1 and Box Forest Road-2 were used to produce maximum dust clouds.

Figure 1 provides details about the roads and a picture of the surface of each road segment.

### Test setups

Data were collected in mobile and stationary test set-ups for vehicle speeds ranging from 10 km/h to 90 km/h depending on safety considerations and the local road rules.

**Fig. 2:**
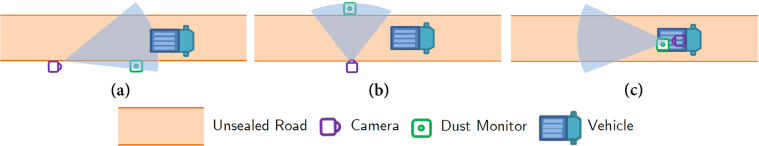
Schematic diagram of test set-ups. (**a**) shows stationary set-up with camera capturing longitudinal view of the road, (**b**) shows stationary set-up with camera capturing side view of the road, and (**c**) shows mobile set-up with camera attached to vehicle capturing rear view while in motion.

**Fig. 3:**
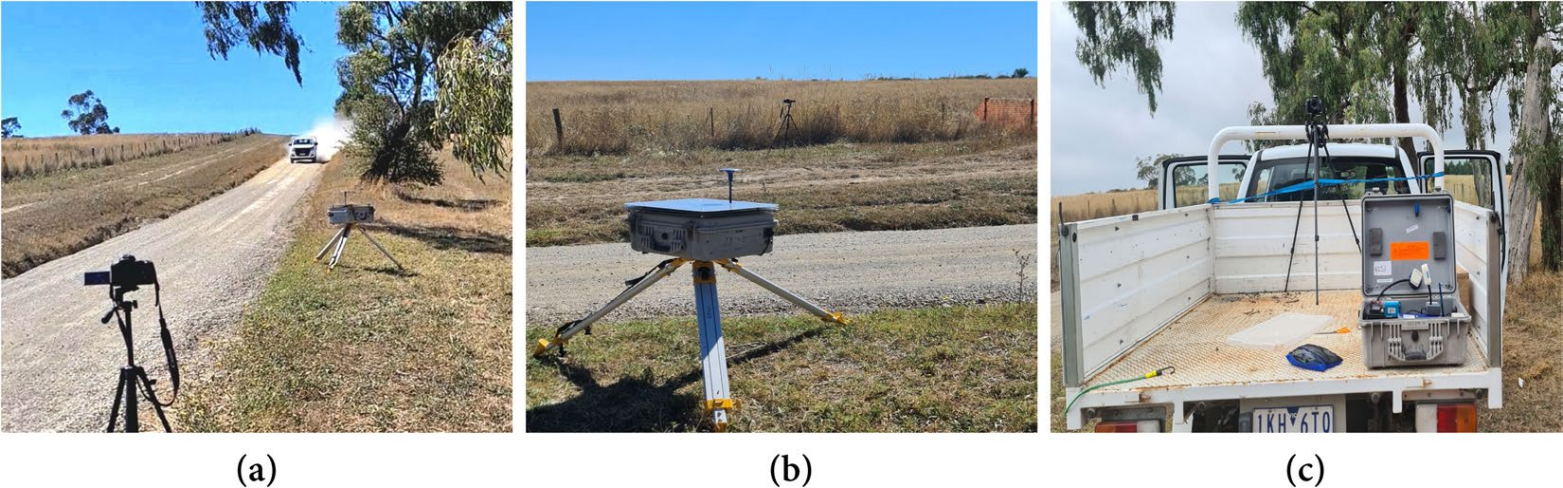
Real arrangement of instruments at sites. (**a**) shows stationary set-up with camera capturing longitudinal view of the road, (**b**) shows stationary set-up with camera capturing side view of the road, and (**c**) shows mobile set-up with camera attached to vehicle to capture rear view while in motion.

In the stationary set-up, the camera and the dust monitor were mounted on the side of the road. Two individual test configurations were applied, depending on the positioning of the camera. The camera either had a longitudinal view of the road or a side view of the road as the test vehicle was driven past it. The two test configurations are shown schematically in Fig. 2a,b. The actual experimental set-ups for the stationary configuration are shown in Fig. 3a,b.

In the mobile set-up, the vehicle was driven along the roads with the camera and the dust monitor attached to the rear, capturing the background of the rear view. The schematic and the actual test set-ups are shown in Figs. 2c, 3c, respectively.

### Dust measurement

A research-grade real-time dust monitor, the DustTrak^TM^ DRX Aerosol Monitor 8533 from TSI, was used in this study. It is able to simultaneously measure both mass and size fraction continuously every second using the light-scattering laser photometric method^31^. Dust measurements in terms of particulate matter (*PM*_*x*_) of different sizes such as *PM*_1_, *PM*_2.5_, *PM*_4_, *PM*_10_ and total particulate matter were obtained from the dust monitor for each experiment. (Note: *PM*_*x*_ refers to particulate matter less than or equal to *x* microns). In future studies, these dust measurements will be used in conjunction with image data to develop a pragmatic tool to quantify vehicle-induced dust from images.

### Test vehicle

**Fig. 4:**
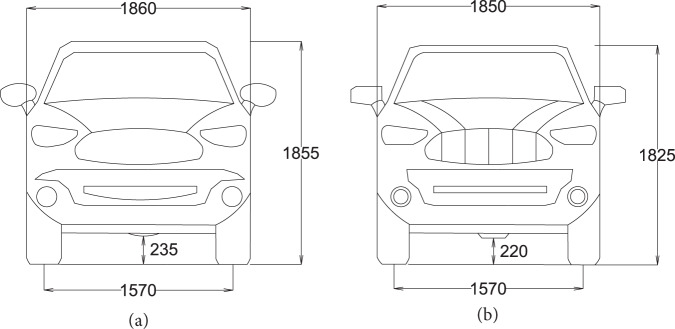
Cross-sections of the test vehicles used for field experiments. (**a**) Isuzu D-Max, (**b**) Nissan Navara ST. All dimensions are in millimeters.

An Isuzu D-Max or a Nissan Navara ST was used for field experiments, and their cross-sections are provided in Fig. 4^32–35^. Other characteristics of the test vehicles are provided in Table 1.

**Table 1:** Properties of test vehicles.

Property	Isuzu D-Max	Nissan Navara ST
Ground clearance unladen (mm)	235	225
Kerb weight (kg)	1860	1944
Overall length (mm)	5295	5120
Aerodynamic drag coefficient	0.47	0.37

### Image collection

The images were collected from videos captured by a Canon EOS 200D direct single-lens reflex (DSLR) camera mounted on a tripod at fixed focal length and 25 frames per second (fps). The camera was positioned according to the test set-up. Multiple angles and positions for the camera were selected to capture most of the dust cloud before it dispersed as well as background features. The dust monitor was placed in the wind-dominant direction so that the dust cloud reached the sampling inlet of the dust monitor. Video recording was started prior to the vehicle beginning to move in order to capture the environment before the dust cloud formed. Therefore, the dataset includes images of the emergence, dissipation and disappearance of the dust cloud, capturing the entire phenomenon. The inclusion of images without dust avoids over-fitting of the dataset.

### Image selection

The videos were captured at 25 fps. We selected one image from every 25 frames in a given second and attributed the selected image to that particular second. The image was selected in order to have a unique data point based on the quality of the image by visual inspection. Images with issues such as blur due to motion, etc. were discarded.

### Annotation

The task of annotation of an image requires that each pixel of the image be labelled as belonging to dust or non-dust. The annotation process starts with converting the selected RGB image to a gray-scale image, dust is then labelled in white and everything else is labelled in black, generating an image with black and white pixels in different shades. Care was taken to detect very minor to stark differences in dust clouds and accurately annotate them, including transparent regions such as the dust cloud boundary and background. Pixel-wise annotation was performed by two mid-level engineers and verified by a senior engineer and a senior academic supervisor. The annotated images were also reviewed by a senior engineer, who is an expert in dust monitoring in unsealed road environments, representing road contractors. It is apparent that there is no definite boundary for dust clouds; however, in the annotation process, we demarcated the boundary visible in the image. The overall annotation process took 1500 + hours.

### Image post-processing

To make manually-annotated images more machine learning-friendly, the images need to be binarized based on pixel intensities. This was done using Otsu’s thresholding method^30^ to generate a new binary image known as a segmentation mask.

Let ${\mathcal{X}}={({{\bf{X}}}_{i},{{\bf{Y}}}_{i},{{\bf{Z}}}_{i})}_{i=1}^{n}$ be a labelled set with *n* number of samples, where each sample (**X**_***i***_, **Y**_***i***_, **Z**_***i***_) consists of an image **X**_*i*_ ∈ℝ^*C* × *H* × *W*^, the corresponding annotated image **Y**_*i*_ ∈{0, 1}^*C* × *H* × *W*^ and its thresholded image **Z**_*i*_ ∈{0, 1}^*H* × *W*^. Each **X**_***i***_ is resized so that the height (*H*) = 1024 pixels, the width (*W*) = 1024 pixels and the class (*C*) = 3 for RGB images. Pixels with 0 and 1 represent the non-dust and dust pixels, respectively. Then, **Z**_*i*_ = *Otsu*(Y_*i*_) where *Otsu*() is the Otsu’s thresholding function.

## Data Records

**Fig. 5:**
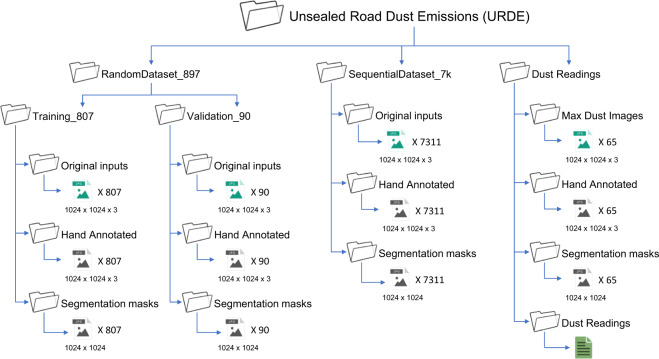
Data repository structure.

The URDE dataset is available from Figshare (10.6084/m9.figshare.20459784)^36^. In the repository, there are three folders, *RandomDataset_897*, *SequentialDataset_7k* and *Dust Readings*. *SequentialDataset_7k* folder has all the images from the experiments and a Google search. *RandomDataset_897* folder has two folders containing 800 images in the *Training* folder and 97 images in the *Validation* folder. The original dataset is *SequentialDataset_7k*. *RandomDataset_897* is a sub-sample of *SequentialDataset_7k*, selected based on the similarity of consecutive images. To minimise undesirable over-fitting phenomena in machine learning and reduce bias, visually similar consecutive images were removed. *RandomDataset_897* is a representative of the larger dataset *SequentialDataset_7k* and it is sufficiently large to segment road dust with high accuracy and produce optimal results. The *Dust readings* folder has dust images corresponding to the maximum dust cloud and a spreadsheet containing the maximum dust concentrations corresponding to that dust cloud. We currently use the dust concentration data to develop a practical dust prediction model for use in the maintenance of unsealed roads. For each image in the folder, an annotated image and a segmentation mask exist in respective sub-folders. *RandomDataset_897* was included so that any researcher could reproduce our results or reduce unnecessarily longer training time upon checking for the trainability of new ML models. *SequentialDataset_7k* is included for future works, in particular, where sequential images are advantageous. Figure 5 illustrates the file structure of the repository.

## Technical Validation

**Fig. 6:**
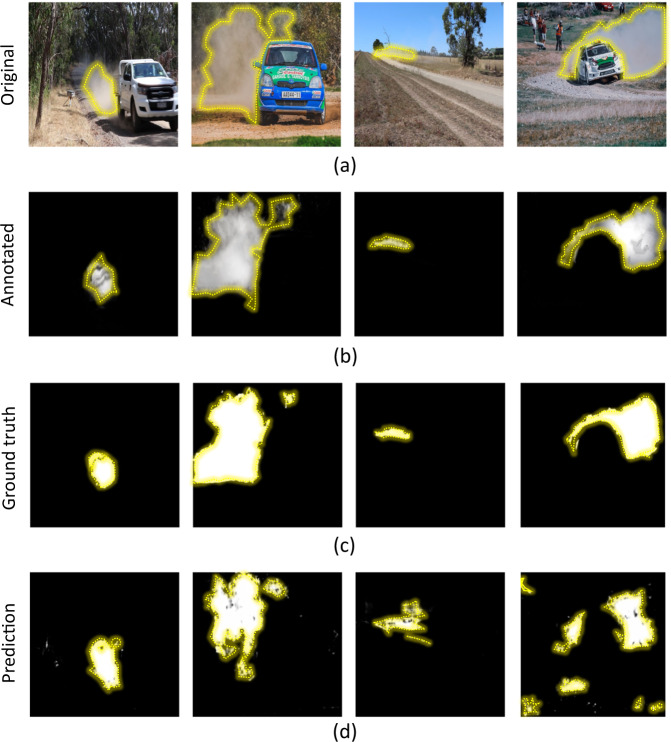
Visualisation of segmentation. (**a**) Original images from the dataset, (**b**) Manually annotated images, (**c**) Images segmented using Otsu’s thresholding, (**d**) Dust cloud predicted by U-Net. Dust cloud boundary is demarcated by yellow dotted lines in all images.

The original images, annotated images and produced secondary images were validated for quality and trainability using the U-net architecture. U-net was selected as it performs moderately well for binary segmentation tasks across multidisciplinary datasets, and it is the root architecture for many modern architectures such as DenseUNet^37^. In Fig. 6, we present qualitative results for our dataset with baseline variables of the architecture and hyper-parameters. We conducted a comprehensive analysis of the dataset for multiple state-of-the-art ML algorithms, and the results showed that the accuracy of segmentation of dust by different ML models increases from the pioneering vanilla Unet to more advanced architectures such as DeepLabV3.

### Segmentation performance evaluation

**Fig. 7:**
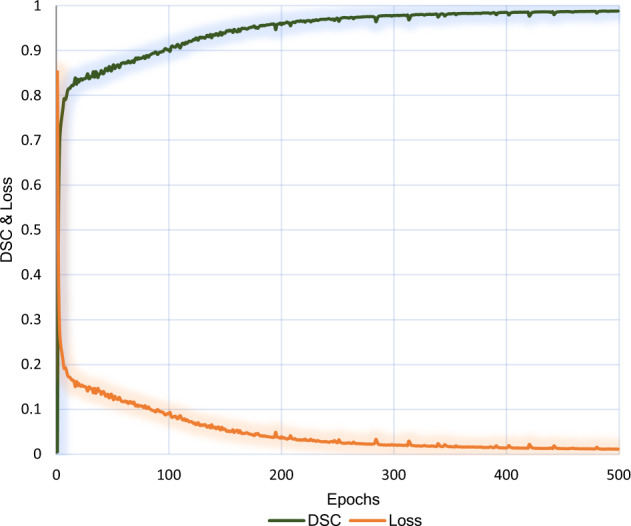
DSC and Loss.

To characterise the results of our experiments, we chose the Dice Similarity Coefficient (DSC)^38^ (Eq. 1) and the Loss. The two parameters are plotted against the number of epochs for the training as shown in Fig. 7. Table 2 shows the quantitative results and hyper-parameters from evaluation. The training platform used for evaluation is described in Table 3.

1 $$DSC(A,B)=\frac{2\cdot | A\cap B| }{| A| +| B| }=\frac{2\cdot TP}{2\cdot TP+FP+FN}$$

where, A and B are sets of voxels of ground truth and the segmented images, respectively. TP, FP and FN are the total number of true positive voxels, false positive voxels and the false negative voxels, respectively.

**Table 2:** Quantitative results and Hyper-parameters.

Segmentation model	Validation DSC	No. of Epochs	Learning rate	Batch size	Model input resolution
U-net	0.906	500	5 × e-4	4	256 × 256

**Table 3:** Training Platform.

	GPU	Video-RAM	System-RAM
Platform	Nvidia 3080 mobile	16GB	32GB
Usage	Fully	5.2GB	11.5GB

For training, the batch size and number of epochs were selected to be 4 and 500, respectively. The batch size of 4 means that four images are processed before the internal model parameters are updated. By making the number of epochs 500, the model does 500 complete iterations through the training dataset. The input layer of the ML model accepts an RGB image vector with 256 × 256 resolution. We selected 256 × 256 resolution conservatively to get the best performance without having a bulkier ML model because high input resolutions exponentially increase the model parameters and subsequently the model size without any added benefit to precision.

### Precision and repeatability of data

In the annotation process, randomly sampled images from the dataset were annotated independently by two annotators. The annotated images were reviewed independently by a senior engineer and a senior academic supervisor. Similar image sets annotated by the two annotators were checked for trainability using U-Net architecture, and the DSC was evaluated for comparison to ensure all secondary data are repeatable.

### Limitations

**Fig. 8:**
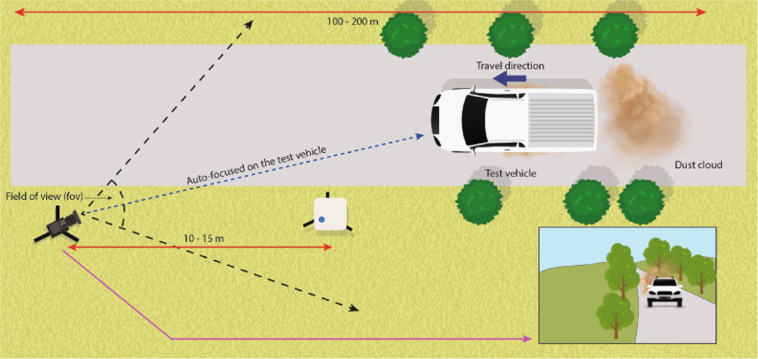
Boundary conditions of the field experiments.

Several limitations of the proposed method were identified as follows:

1. The dataset contains images where the background or the road in the image does not complement the appearance of the dust cloud due to its visual similarity. This results in a “camouflage effect” where the model is unable to distinguish between the dust cloud and the background or the road. Therefore, a part of the background or the road may be misrecognised as dust.
2. A majority of the images in *RandomDataset_897* folder includes ~800 images collected from experiments conducted in Victoria, Australia. The 897 images include 85 copyright-free images from Google, 189 images from Hughes road, 219 images from Muir road, 209 images from Wallace road, 112 images from Ryans lane and 83 images from Finch road, respectively. The 85 copyright-free images were added to increase variability. However, due to the disproportion between the number of images from experiments and the images from Google, the model currently fits best for unsealed roads in Australia.
3. In the binarizing step, the regions where the dust cloud was transparent are identified as dust pixels leading to over-estimation of dust cloud from the image.
4. To ensure the precision and repeatability of the data collected in field experiments, we established experimental parameters and boundary conditions shown in Fig. 8. The Canon EOS 200D DSLR camera was set to auto-focus the oncoming vehicle (Isuzu D-Max or a Nissan Navara ST). The camera position and field of view were selected so that 100 to 200 meters of road length is visible in the video. The dust monitoring device (DustTrak^TM^ DRX Aerosol Monitor 8533 from TSI) was positioned 10 to 15 meters from the camera. The resolution of the video and image was set to 1920 × 1080 pixels. The traffic-induced dust emission in unsealed roads is affected by many factors, which include wind speed and direction, condition of the surface material, vehicle tire type and its condition, etc.; however, similar data could be reproduced if the field experiments are conducted with the experimental parameters and boundary conditions shown in Fig. 8.

Limitations 1 and 2 may be resolved by expanding the URDE dataset, which is publicly available at 10.6084/m9.figshare.20459784. Limitation 3 may be resolved using a different thresholding technique.

## Usage Notes

The dataset includes original images, manually-annotated images and the corresponding binary images generated by Otsu’s thresholding method. The binary images generated from Otsu’s method can be obtained using the Python-based tool included at https://github.com/RajithaRanasinghe/Automatic_Thresholding. The set of original images together with their corresponding binary images may be used in conjunction with machine learning architectures to train the dataset and predict dust in an image. The dust measurements from the dust monitor may be used to correlate the dust cloud with actual dust readings. The process may be extended to videos and real-time dust prediction applications.

## Data Availability

All the Python scripts used to generate the secondary data (binary images by Otsu’s thresholding) are provided at https://github.com/RajithaRanasinghe/Automatic_Thresholding.
